# Lessons learned from lung and liver in-vivo gene therapy: implications for the future

**DOI:** 10.1080/14712598.2018.1506761

**Published:** 2018-08-10

**Authors:** Joost van Haasteren, Stephen C. Hyde, Deborah R. Gill

**Affiliations:** Gene Medicine Group, Nuffield Division of Clinical Laboratory Science, Radcliffe Department of Medicine, University of Oxford, Oxford, UK

**Keywords:** Gene therapy, in-vivo, ex-vivo, lentivirus, adenovirus, AAV, gene editing, pseudotype, non-viral vector, cystic fibrosis

## Abstract

**Introduction**: Ex-vivo gene therapy has had significant clinical impact over the last couple of years and in-vivo gene therapy products are being approved for clinical use. Gene therapy and gene editing approaches have huge potential to treat genetic disease and chronic illness.

**Areas covered**: This article provides a review of in-vivo approaches for gene therapy in the lung and liver, exploiting non-viral and viral vectors with varying serotypes and pseudotypes to target-specific cells. Antibody responses inhibiting viral vectors continue to constrain effective repeat administration. Lessons learned from ex-vivo gene therapy and genome editing are also discussed.

**Expert opinion**: The fields of lung and liver in-vivo gene therapy are thriving and a comparison highlights obstacles and opportunities for both. Overcoming immunological issues associated with repeated administration of viral vectors remains a key challenge. The addition of targeted small molecules in combination with viral vectors may offer one solution. A substantial bottleneck to the widespread adoption of in-vivo gene therapy is how to ensure sufficient capacity for clinical-grade vector production. In the future, the exploitation of gene editing approaches for in-vivo disease treatment may facilitate the resurgence of non-viral gene transfer approaches, which tend to be eclipsed by more efficient viral vectors.

## 1. Introduction

Gene therapy holds a major promise: to provide a cure for genetic diseases that often lack a practical and realistic treatment. While genetic diseases are inherited, and thus the mutation is present in all cells, disease pathology often manifests in only certain tissues or organs. Diseases such as cystic fibrosis (CF) and primary ciliary dyskinesia (PCD) display their primary phenotype in the lung, where the protein involved is normally expressed, and thus the cells in the lung will be well suited to gene therapy for these kinds of diseases. The liver, an organ crucial in the production of secreted serum proteins, is the main target for gene therapy to address diseases such as hemophilia. Alpha-1 antitrypsin (AAT) is produced in the liver, but a lack of the secreted AAT can cause lung emphysema. Reports of AAT expression in the lung being beneficial make both the lung and liver viable targets to treat this disease. Gene therapy generally involves the transfer of a nucleic acid construct that expresses a functional copy of a gene into a cell, to supplement a missing or mutant protein. Significant progress has been made, however, in genome editing where the mutation is corrected directly in the patient’s genome. Despite early setbacks [1], gene therapy has made important progress over the last decade [2]. For in-vivo application, the delivered nucleic acid is injected directly into the patient. This often happens via the use of a carrier (or vector), which can consist of lipid or polymer to protect the nucleic acid cargo, or by exploiting viruses that are expert in injecting nucleic acids into cells. In recent years, the bulk of effort in gene therapy development has rightly focused on improving vectors to deliver the therapeutic nucleic acids to the target cells. In this review, we discuss the in-vivo gene therapy approaches taken to effectively deliver and express therapeutic transgenes and/or gene editing machinery in the lung and liver. We also look at the progress made in ex-vivo gene therapy applications, how we can apply this knowledge to in-vivo gene therapy and review both potential challenges and exciting possibilities for the future.

## 2. A brief history of in-vivo gene therapy

### 2.1. Delivery, delivery, delivery

The key challenge for successful gene therapy is to deliver sufficient quantities of the therapeutic nucleic acid payload into the right cell. The *right* cell is usually the cell type in which the targeted protein is normally produced in healthy individuals. Alternatively, a cell type where production of said protein can also be beneficial, for instance delivery to the muscle to produce secreted proteins such as AAT, is sometimes referred to as a ‘protein factory’ [3]. Ensuring the gene therapy vector expresses the therapeutic transgene in the right cell can be difficult, as systemic delivery in particular, but also topical delivery to some degree, will widely dissipate the vector through (parts of) the body via the lymphatic system or blood circulation. Thus, gene delivery requires a reasonable amount of precision, not only in targeting the correct cell but to avoid non-target cells. Developing a successful single-organ delivery strategy, suitable for all applications in that particular organ, is unlikely because diseases affecting the same organ can originate from different cell types. The following sections set out the ways in which the gene therapy field has developed delivery approaches that fulfill these requirements for lung and liver gene therapy and how each organ poses its own challenges and opportunities.

A successful in-vivo gene therapy must be developed using a three-pronged approach, focusing on the delivery method, the delivery vector, and delivery to target cells.

### 2.2. Lung

#### 2.2.1. Delivery method

Gene therapy for the lung has garnered much attention over the years, as lung delivery was once seen as relatively straightforward and therefore targeting lung disease was considered ‘low-hanging fruit’. In particular, the discovery of the gene for the common, recessive, genetic disorder Cystic Fibrosis (CF) in 1989 [4] has been an important driver for lung gene therapy.

**Figure 1.**
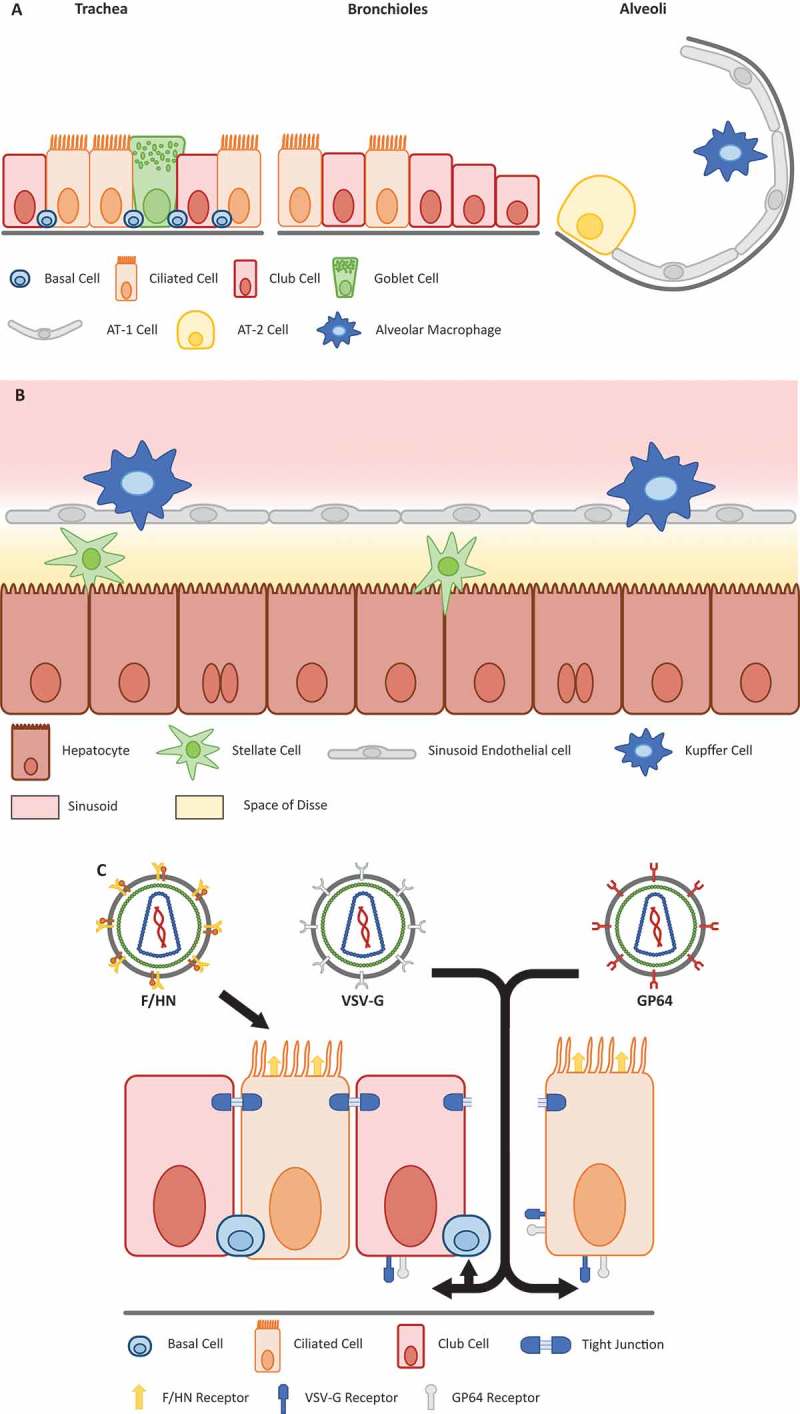
Schematic of main cell types in the lung and liver. **(a)** The lung can roughly be divided into three compartments proximally to distally: the trachea, bronchioles and alveoli. Ciliated cells are mainly present in the trachea and bronchioles, and are the key target cell for gene therapy for primary ciliary dyskinesia. For CF, the ciliated cells primarily lining the bronchioles are targeted. Basal cells represent one of the purported stem cell niches and are therefore an important target for gene therapy. The location of basal cells below the ciliated and club cells of the stratified epithelium, means that some form of mechanical or chemical cell junction disruption is required to access them. The alveolar type 2 (AT-2) cells are the main producers of surfactant proteins and thus a target cell type for gene therapy for surfactant deficiencies. (**b)** In the liver, the predominant target cell is the hepatocyte, which are the main source of many proteins in the blood such as albumin. Hence, they constitute an important target for the production of secreted proteins such as alpha-1 antitrypsin and clotting factors VIII and IX. Both Kupffer cells and stellate cells have reported antigen presentation capacity, which suggests that transduction and expression in such cells is to be avoided to prevent unnecessary immune responses to transgene product or viral proteins. (**c)** Location of viral receptors on polarized cells of the lung epithelium can affect in-vivo applications. The receptor for the lentiviral F/HN pseudotype is directly accessible via the apical surface, whereas receptors for VSV-G and GP64 pseudotypes may be located more basolaterally, requiring the addition of chemical adjuvants for efficient airway gene delivery. A similar strategy is required to access the basal cells of the epithelium.

The primary route for lung gene transfer has been direct delivery via the airways, typically through the generation of respirable aerosols; this is a tried and tested approach for the delivery of multiple conventional therapeutics for a wide range of lung disorders. In this way, the lung epithelial cells are directly accessible (see Figure 1(a)) and the possibility of the gene therapy vector targeting other tissues and organs is reduced. Aerosolization is possible for a range of gene therapy delivery vectors with varying degrees of success [5], although this does require formulation development to protect vectors that are vulnerable to shear forces generated by nebulizers. Promising results have been achieved with the more gentle, vibrating mesh nebulizers, albeit to-date with protein replacement therapy rather than with a gene therapy vector [6].

Intravenous (IV) injection, also known as systemic delivery, has also been investigated as a route to deliver a gene therapy to the lung via the vasculature, but tends to result in delivery to lung endothelial, rather than epithelial, cells. Using a novel nanoparticle with a combination of lipids, however, both endothelial and epithelial cells (CD326^+^CD45^−^ cells) have been targeted in mouse lung [7]. It is important to note that while this study reported ~34% delivery to the lungs, the majority of the gene therapy agent inadvertently ended up in the liver.

In early clinical trials of lung gene therapy, the nasal epithelium has often acted as a surrogate tissue, as it also features ciliated cells, and being more accessible than the lung it is more readily available for biopsy and harvesting of cells via nasal brushing. Intranasal (IN) delivery also requires a smaller gene therapy vector dose due to the smaller surface area compared with the lung. In CF in particular, access to the nasal epithelium allows direct measurement of ion transport defects thereby reporting successful restoration of expression of the Cl^−^ channel cystic fibrosis transmembrane conductance regulator (CFTR) [8]; such measurements are more invasive and difficult in the lung, usually requiring a general anesthetic [9].

Direct injection into the pleural space, sometimes regarded as an ‘outside-in’ approach to lung delivery, is another promising administration route. An ongoing phase I/II clinical trial using a recombinant adeno-associated virus (rAAV) vector to express AAT for treatment of AAT deficiency will inform the applicability of this delivery method [10].

#### 2.2.2. Delivery vector

One of the first extensively studied vectors for lung gene therapy was recombinant adenovirus (rAd), but promising results observed in animal models were not recapitulated in early clinical trials, casting doubt on the efficacy of rAd in the human lung; transgene expression from rAd, although robust, was transient, likely due to a strong CD8^+^ cytotoxic T-cell response to adenovirus genes expressed in the transduced cells [11]. One way to circumvent this issue is to completely avoid the inclusion of viral coding-regions or proteins in the vector, focusing instead on non-viral mediated gene delivery; this approach continues to be extensively studied for gene therapy in the lung. Advantages of non-viral vectors include an almost unlimited packaging capacity, a good safety profile, the ability to package RNA species as well as DNA, and the ability to be successfully repeat administered. Lacking the signals necessary for an adaptive immune response (i.e. proteins), non-viral vectors benefit from low immunogenicity, although the DNA can still elicit an innate inflammatory response mediated via Toll-like receptor 9-dependent CpG recognition [12]. This can be tackled by the removal of all CpGs from the plasmid vector, resulting in reduced inflammation and considerably increased duration of transgene expression [13]. Crucially, the lack of an adaptive immune response allows for effective repeat administration of non-viral vectors, as observed in clinical trials for CF in both the nose [14] and lung [15]. In the latter study, monthly aerosol administration of DNA/liposomes was sufficiently effective to produce significant improvements in lung function. Non-viral vectors are usually based on cationic lipids [16,17] or polymers [18], encapsulating plasmid DNA or siRNA species. Unlike most viral vectors, non-viral vectors lack an active mechanism to import their genome into the nucleus of targeted cells, although passive import features can be added by the incorporation of tissue-specific DNA nuclear import signals found in certain promoter sequences, including the lung-specific SP-C promoter [19]. Non-viral vectors can also be combined with targeting peptide moieties to promote cell specificity and targeting [17], although in doing so, some of the advantages of a non-viral vector begin to be eroded.

Similar to rAd vectors, gene delivery to the lung epithelium via recombinant Adeno-associated virus (rAAV) looked promising in animal models but translation to humans has fallen short of expectations in terms of transgene expression [20]. Early trials mainly utilized AAV serotype 2 [21,22], where the main AAV2 receptor (heparin sulfate proteoglycan) and one of the main (co)receptors of AAV2 and other serotypes (AAVR) are located on the basolateral membrane of polarized epithelial cells in humans, limiting direct access for rAAV from the treated patients lung lumen [23,24]. Similar issues arise with commonly used pseudotypes of recombinant lentivirus (rLV). The most widely used rLV pseudotype utilizes the envelope glycoprotein from the vesicular stomatitis virus, the canonical VSV-G pseudotype. Successful transduction of lung epithelial cells with VSV-G requires pre-treatment with adjuvants to open the tight-junctions between epithelial cells, or to strip away the epithelial layer completely, so that the rLV can access receptors on the basolateral surface and basal cells respectively (Figure 1(c)) [25]. Given the greatly increased risks of infection and sepsis following such manoeuvres, these approaches are unlikely to be directly translated into the clinic.

#### 2.2.3. Delivery to target cells

The lung contains multiple cell types that require targeting with gene therapy vectors in order to treat different diseases (Figure 1(a)). In CF, lung disease begins in the small airways where the CFTR protein is primarily expressed [26,27]. In contrast, for treatment of surfactant protein deficiencies, the target cells are located in the parenchyma, mainly in alveolar type 2 (AT-2) cells. The spatial distribution of the cells within the lung is also important. For example, in PCD the ciliated cells of the trachea and primary bronchi are more affected, compared with the more distal cells affected in CF. While targeting stem cells within the lung would be advantageous, there is currently poor consensus on the identity and location of the lung stem cell population [27] and compelling evidence that each lung compartment has its own progenitor cell type and niche [28]. Thus, there is currently no single lung stem cell that can be targeted for all disease applications.

##### 2.2.3.1. Tropism and pseudotyping

**Figure 2.**
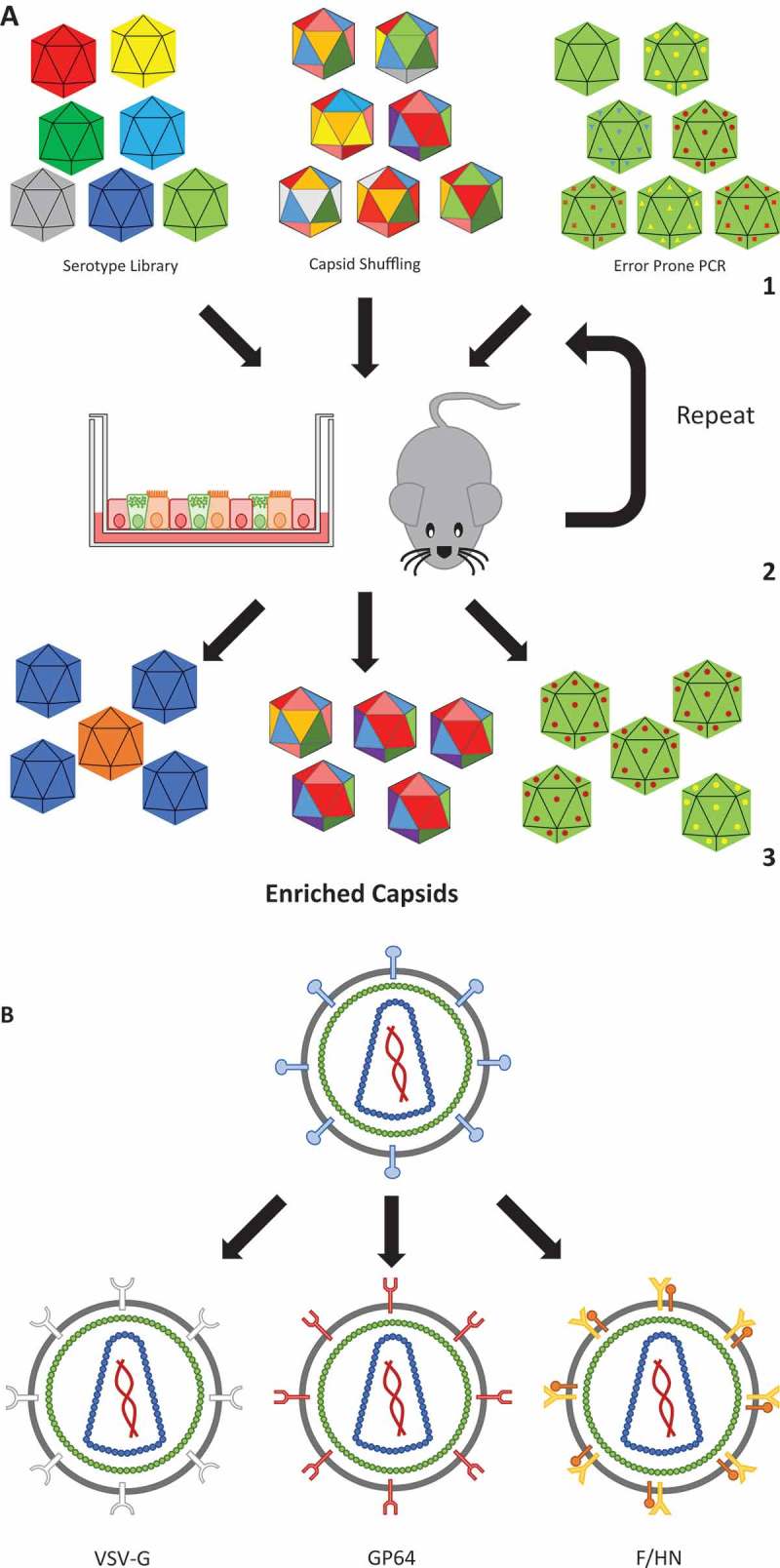
AAV capsid evolution and lentivirus pseudotyping. **(a)** Directed viral capsid evolution is commonly used to engineer novel capsid variants that are efficient in the transduction of a very specific cell type or tissue. To achieve this, a library of serotypes are used, which could consist of already known serotypes, or novel ones containing random parts of other serotypes (capsid shuffling), or capsids derived by error-prone PCR amplification that usually results in one to three point mutations (1). These libraries are used to transduce a target which can be in-vitro (e.g. an air-liquid interface culture) or in-vivo (e.g. a humanized mouse). Subsequently, the viral genomes are extracted and used to create a new batch of virus (2). Repeating this results in the enrichment of virus variants that are proficient in transducing the target cells (3). The same strategy can also be employed for the evolution of envelope proteins for enveloped viruses. (**b)** Retroviruses and lentiviruses have their receptor binding proteins (e.g. gp120 and gp41 in the case of HIV1) located on their viral envelope. Such receptor binding proteins can be replaced by envelope proteins to target a desired cell type (for instance the multi-tropic VSV-G or lung-tropic F/HN), via a process called pseudotyping.

Vector engineering, and particularly directed evolution whereby specific characteristics can be selected (detailed in Figure 2(a)), has the ability to expand the cell tropism for vector targeting. This approach used on human airway epithelial cultures has benefitted the development of rAAV vectors resulting in increased transduction efficiencies in the lung [30–32]. It is interesting to note that the novel viral capsids that are most successful are often made up from large parts of capsid sequences derived from Clade A viruses, which already display improved lung epithelium transduction compared to other clades [31,32].

The generation of helper-dependent adenoviruses (HD-Ad) has led to a revived interest in the use of adenoviral approaches generally, since these vectors are completely devoid of virally encoded genes, thereby drastically reducing their immunogenicity in-vivo. This reduction is the likely reason that transgene expression is more persistent than that observed for early rAd vectors, but as the reduction is not absolute the risks of immune system surveillance increase upon re-administration which might result in cessation of transgene expression [33]. Encouragingly, recent work has shown that HD-Ad can be used to successfully transduce respiratory tissue in mice, pigs and human airway cultures [34]. Interestingly, in these studies, it was the basal cells that were the main target (see Figure 1(a)), which have been reported to have progenitor functions and as such are likely to also contribute to the observed improved duration of expression.

**Figure 3.**
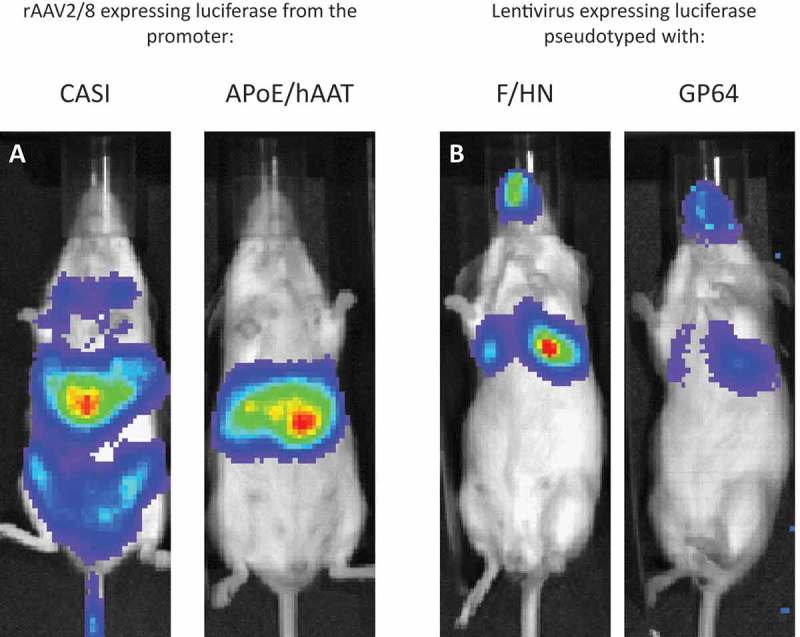
Pseudotype and promoter choice can influence transgene expression. **(a)** Mice were administered with a similar dose (1e11 genome copies) of rAAV2/8 injected intravenously and imaged 7 days post-dosing. The only difference is the choice of promoter to drive luciferase reporter expression. CASI is a ubiquitous promoter and shows that rAAV8 transduces the liver but also other parts of the mouse. Replacing CASI with the liver-specific APoE/hAAT promoter maintains strong expression in the liver and largely prevents expression in off-target cells. (**b)** Mice were administered with a similar dose (1e8 transducing units) of the same rLV vector configuration expressing luciferase reporter gene, but pseudotyped with either F/HN or GP64, and were live imaged 7 days post-dosing. Increased transduction efficiency can be explained in part by the distribution of receptors. F/HN has direct access to its receptors (sialic acid) on the apical surface of the epithelium. Binding to GP64 (and VSV-G) receptors requires pre-treatment (1% methylcellulose used here) that may help access receptors on the basolateral membrane of the polarized epithelium (see Figure 1(c)).

Poor lung transduction rates observed with the canonical VSV-G pseudotype led to the development of novel lentiviral pseudotypes with improved transduction efficiencies (reviewed in [35]). A lentiviral pseudotype based on the envelope proteins of the lung-tropic Sendai virus (Figure 3(b)) has shown promising results in pre-clinical models and is poised for clinical trial [36,37]. A novel lentiviral pseudotype, derived by error-prone PCR-mediated directed evolution (Figure 2(a)) of the basic GP64 pseudotype, improved expression in human primary airway epithelial cells [38]. Interestingly, the transduction efficiency of the derived pseudotype declined in porcine airway epithelial cells, indicating that the directed evolution had favored receptors expressed primarily in humans. This also exposes a limitation of experiments with reagents specifically optimized for human tissue delivery where currently available animal models might not be sufficiently transduced to permit meaningful dose and toxicity studies in preparation for clinical trials.

##### 2.2.3.2. Restricting expression

Due to the nature of the preferred vector delivery method in lung gene therapy, nebulization directly into the airways, the chance of accidentally transducing unwanted tissues or organs is reduced. This typically permits the use of ubiquitous promoters, which are often stronger than their tissue-specific counterparts (see Figure 3(a) for an example of a liver-specific promoter APoE/hAAT compared with the ubiquitous CASI promoter). However, reports suggest that combining the CASI promoter with liver-tropic rAAV serotype 8 leads to transduction of the lung, and also the liver, after intra-tracheal delivery [39]. This suggests that in the development of a suitable gene therapy product, careful selection of the genomic construct and vector combination is required, even in a restricted delivery organ such as the lung. There are several lung cell type-specific promoters characterized, some of which are based on the promoter regions of the surfactant protein family [40–42]. Surfactant protein B (SP-B) could be deleterious when expressed in cells that do not express the necessary enzymes to process the precursor versions of SP-B [43]; this makes the use of an AT-2 cell-specific promoter such as SP-B or SP-C advantageous. Using a viral carrier such as rAd, with a greater payload capacity, allows large promoters such as cytokeratin 18 to be utilized [34].

##### 2.2.4. Lung specific challenges and opportunities

Despite the relative ease of access to the lung epithelium, there are several physical and chemical barriers to vector delivery [44]. In addition, specific diseases causing reduced ciliary motility (as in PCD) or changes in mucus quality such as viscosity (as in CF) can cause a buildup of mucus in the lung, constituting a physical barrier to lung delivery, and trapping the vector before transduction of the target cell can occur. Furthermore, mucus buildup is often associated with inflammation, which can lead to reduced efficacy of viral vectors when they are taken up by an increased number of resident lung macrophages, attracted by a boosted immune system. Both these issues can possibly be overcome by mucus-penetrating nanoparticles, or the co-administration of mucolytic drugs [45].

As mentioned before, alternate genetic lung diseases may originate from different cell types and in different regions of the lung [26,27]. This represents a challenge to develop platform gene therapies for multiple diseases, since both the vector tropism and delivery method will have to be fine-tuned to meet specific disease needs. Thus, the development of specific clinical products, refined for each lung disease, is common; an approach which is both more time-consuming and costly.

One of the main challenges of gene therapy, translating findings from pre-clinical models to the clinic, is particularly difficult in the lung. The mouse lung differs from humans not only in the types of (viral) receptors expressed, but also their distribution within lung compartments. Alpha-2,6 sialic acid-linked receptors, for example, are expressed in the more distal parenchyma of the lung in mice but more proximally, in the trachea, in humans; while the reverse is true for alpha-2,3 sialic acid-linked receptors [46,47]. Receptor expression also differs with regards to the location on the cell membrane. For instance, the receptor for adenovirus serotype 5 (the coxsackie and adenovirus receptor CAR) is expressed apically in mice (and thus easily accessible to topical vector delivery), but basolaterally in humans [48,49]. This disparity in receptor distribution likely underpins the failure of rAd in human trials to deliver on the early successes in murine studies.

In mice, the ciliated cell that is the target cell for both CF and PCD lasts only about 6 months in the trachea and up to 17 months more distally in the lung, and is unlikely to persist significantly longer in humans [50]. This means that theoretically any single treatment will no longer be effective after that time, unless stem cells are stably transduced (i.e. there is a copy of the gene integrated in the genome by for example using a lentivirus). As mentioned before, the identities of lung stem or progenitor cell are contentious and designing vectors to target those cells specifically will require a trip back to the drawing board.

### 2.3. Liver

#### 2.3.1. Delivery method

Early approaches to genetic treatment of the liver involved culturing a liver biopsy ex-vivo, transducing it with a recombinant retrovirus (rRV) and subsequently infusing it into the inferior mesenteric vein to allow re-engraftment of the transduced cells into the patients’ liver; however, this rather complicated gene/cell therapy approach has not progressed [49]. However, similar to lung, the anticipation of a straightforward route of administration, specifically via intravenous infusion, has brought liver gene therapy to the forefront of the in-vivo gene therapy field. Indeed, the primary function of the liver in the body, filtering blood, implies that a significant portion of any gene therapy agent that is infused intravenously will eventually end up in the liver. Furthermore, non-clinical studies for liver gene delivery have benefitted from the use of hydrodynamic (tail vein) injection to mice, a highly efficient delivery strategy, which requires the injection of a relatively high volume of liquid carrying the gene therapy vector. This high volume (up to 10% of body weight in rodents) causes a congestion in the right ventricle allowing retrograde flow of the gene therapy liquid back through the portal vein [52] at significant pressure. It is this transient increase in hepatic pressure that ensures efficient vector delivery throughout the organ. This method even allows for the use of the simplest form of gene therapy vector: naked DNA (i.e. DNA, typically in the form of a bacterial plasmid, without any coating or lipid complexation). Occluding the hepatic artery can aid in this process, especially in larger animals by providing additional back-pressure within the liver further enhancing vector delivery. Interestingly, using a virus with a known hepatic tropism, and promoter-mediated cell de-targeting, Greig et al. showed that even a topical intra-muscular (IM) injection can lead to liver-restricted expression in mice, confirming that in gene therapy, most roads lead to the liver [53].

#### 2.3.2. Delivery vector

Multiple vectors have been used for liver gene therapy in non-clinical models, due at least in part to the straightforward delivery methods available for in-vivo liver delivery. Non-viral vectors can simply be injected via the tail vein in rodent models, with many innovations developed to increase transgene expression by minimizing the bacterial backbone of plasmid DNA, including the use of minicircles and mini-intronic plasmids [54,55]. While non-targeting vectors have been used successfully to transduce hepatocytes, using a virus with a defined hepatocyte-specific tropism can both improve specificity and efficacy. In liver gene therapy, it is rAAV that is most widely used, particularly in clinical trials. Several wild-type AAV serotypes display inherent hepatic targeting (see also Delivery to target cells below) and their non-pathogenic track record makes them ideally suited for in-vivo gene therapy. However, other viruses have been successfully used in non-clinical models, including rLV [56], HD-Ad [57] and exosome-enveloped rAAV [58].

#### 2.3.3. Delivery to target cells

Many viruses have a natural tropism for the liver and will, due to the IV delivery method, come into contact with hepatocytes making it highly likely that these will be transduced to some degree (see Figure 1(b)). However, it is paramount that for effective liver gene therapy it is only the hepatocytes that are transduced and that they are transduced to a degree that allows therapeutic transgene expression. This is to avoid adverse immune responses, due to transduction of resident antigen presenting cells (APCs) in the liver (Kupffer cells and hepatic stellate cells). Therefore, there has been much effort in (i) developing new AAV serotypes and lentivirus pseudotypes adept at transducing hepatocytes but avoiding other cell types; and (ii) developing vector constructs that promote hepatic expression while minimizing expression in non-hepatic cells that are transduced inadvertently.

##### 2.3.3.1. Tropism and pseudotyping

The AAV8 serotype is regarded as the ‘canonical’ liver-tropic AAV serotype, as evidenced by the many non-clinical and clinical experiments where AAV8 has been center stage (see Figure 3(a) for an example of rAAV2/8 liver tropism in mice). Recombinant AAV8 was the vector used in the first liver-targeted clinical trial for hemophilia B [59], although this pioneering trial once again proved that results obtained in animal models rarely translate directly to humans. The trial saw an initial spike of factor IX (FIX) expression wane over time due to an anti-AAV8 capsid protein cytotoxic T-cell response, something not seen in animal experiments. Additionally, while rAAV8 mediates highly efficient liver transduction in mice, human liver is less efficiently transduced, leading others to favor other serotypes such as AAV5 and the use of hyperactive protein variants such as the Padua mutation of FIX [60,61]. Not satisfied with naturally occurring serotypes, others have used humanized mice (where mouse liver cells are partially replaced with human liver cells) to allow directed evolution of AAV capsid (Figure 2(a)), developing novel serotypes that are better at targeting human cells [62,63]. Similar to the directed evolution approach in human airway epithelial cultures described above, when these novel serotypes increase their tropism for human liver cells, they concomitantly lose tropism for mouse cells. This limitation makes it difficult to extrapolate non-clinical data to the clinic, especially if cumbersome and expensive humanized mice are not widely available. This has prompted others to consider human liver organoids as a possible intermediate step or substitute tissue for capsid selection [64].

Besides rAAV, other viral vectors that have attracted interest in the liver gene therapy field include retro- and lentiviruses. The well-established VSV-G pseudotype has a very broad tropism and is able to transduce the liver when injected directly into the bloodstream, but as in the lung (Figure 3(b)) replacing VSV-G with alternative pseudotypes can lead to changes in both efficacy and specificity. The baculovirus-derived GP64 protein appears to display greater liver tropism compared with VSV-G, which leads to more significant transduction and higher transgene expression profile in mice [65,66]. Other pseudotypes, such as those derived from alphaviruses Ross River virus or Semliki Forest virus, are able to efficiently transduce both hepatocytes and Kupffer cells [35]. Transduction of these cells, which can exhibit antigen display properties, could be detrimental through the induction of a cellular immune response against both viral structural proteins and the therapeutic transgene protein, and should be avoided.

Researchers have also managed to deliver therapeutic microRNAs to hepatocytes by tagging the oligonucleotides with N-acetylgalactosamine, making it efficiently endocytosed by the hepatocyte-specific asialoglycoprotein receptor [67]. This shows that, similar to advances in viral gene therapy, non-viral vectors can also be improved with a ‘pseudotype’ to increase their efficacy and specificity.

##### 2.3.3.2. Restricting expression

Even when care is taken to select a vector that exhibits highly specific liver tropism, it appears that some off-target transduction cannot be avoided and using a ubiquitous transgene promoter (e.g. CMV or CASI) is inadvisable for this reason alone. While such promoters may display robust expression, they typically direct transgene expression in every transduced cell, which in many settings could be detrimental (see Figure 3(a) showing widespread expression with the ubiquitous CASI promoter). Similarly, there are reports of loss of expression over time when using ubiquitous promoters, most notably CMV [68]. There are examples of promoters used in non-clinical and clinical experiments that utilize enhancer/promoter regions from proteins that are exclusively expressed in hepatocytes, such as apolipoprotein A, AAT, transthyretin and thyroxine-binding globulin [53,59,69]. One chimeric enhancer/promoter, termed APoE/hAAT which contains a portion of the hepatic control region of the apolipoprotein A enhancer and a region of the human AAT promoter, has been used in clinical trials successfully [59,70]. These promoters owe their specificity to the incorporation of transcription factor binding sites that correspond to transcription factors expressed exclusively in the liver. Exploiting this knowledge, researchers have used in-silico analysis methods to derive synthetic promoters that are both strong and liver specific [71]. Interestingly, analysis of the ITR region of the AAV2 genome (often used as the genome for many different AAV pseudotypes) has revealed that it contains transcription factor binding sites for, among others, hepatocyte nuclear factor 1 homeobox A (HNF1α) [72]. This transcription factor is strongly liver-specific and, while possibly beneficial or at least neutral in liver-directed gene therapy, could conceivably be detrimental when using an AAV2 genome to target a different tissue or organ, by prompting off-target expression in the liver.

While not common, promoters driving microRNA expression can also be used to drive transgene expression. Due to their regulatory nature, microRNAs are often tissue specific and with that knowledge miR-122 can be used as a hepatocyte-specific promoter [73]. As one might expect, the expression of a hepatic upregulated microRNA, miR-122a, can also be exploited in the reverse fashion, namely by inserting a microRNA target site in the 3ʹ UTR region of the gene therapy transgene, effectively preventing transgene expression in hepatocytes but retaining expression in the sinusoidal endothelial cells [74]. The same group used another microRNA target site, 142-3pT, to effectively prevent expression in transduced APCs, thereby avoiding immune responses caused by antigen presentation of the expressed transgene [75].

#### 2.3.4. Liver specific challenges and opportunities

One intriguing observation regarding liver gene therapy is that expression of a ‘non-self transgene’ can elicit immune tolerance to that protein product [76]. This is important for genetic liver diseases where a mutation causes complete cessation of expression of the mutated gene (e.g. in hemophilia B) and where enzyme replacement treatment often induces inhibitory antibodies to the protein. It has been shown that strong hepatocyte-specific transgene expression, whilst circumventing either the transduction of, or expression in, APCs, can also prevent and even abrogate the formation of such inhibitors [77,78].

One possibly troubling aspect of using AAV for liver gene therapy is the suggestion that the integration of rAAV genomes can contribute to the formation of hepatocellular carcinoma (HCC). Donsante and colleagues found in mice that rAAV2 integration can occur in a 6kb region of chromosome 12, encoding for several microRNAs and small nucleolar RNAs. In tumors found post-treatment, these RNAs were strongly overexpressed, implying that rAAV integration played a causal role [79]. The observation that the internal transgene promoter was not necessary for the increase in tumor incidence is corroborated with a more recent study that noticed the same effect when even only a portion of the viral ITR was integrated [80]. The fact that a region of some rAAV2 ITRs contains a bidirectional binding site for a strong liver-specific transcription factor (HNF1α) aids the speculation that unforeseen integration of AAV ITRs can influence the expression of neighboring genes, similar to the situation observed following retroviral integration [72,81]. It is important to acknowledge, however, that larger animal models have not presented with rAAV-associated HCC and that so far no similar cancers have been reported in the numerous rAAV liver gene therapy clinical trials that have been undertaken [82,83]. Moreover, most humans are seropositive for AAV2, indicating that they have tolerated an AAV2 infection in the past, and to-date, there is no reported evidence that wild-type AAV2 infection correlates with an incidence of HCC [82].

## 3. Lessons learned from ex-vivo success

Contrary to in-vivo gene therapy, the target cells in an ex-vivo gene therapy approach are transduced outside of the patient’s body. The uptake of this strategy has gathered speed over the last decade, especially for primary immunodeficiencies. In these disorders, the hematopoietic stem cells are taken from the patient’s body and transduced, often with integrating viral vectors such as rRV and rLV and transplanted back into the patient. This is highly successful, as corroborated by recent regulatory approvals for adenosine deaminase (ADA)‐deficient severe combined immunodeficiency (SCID), and can provide a life-long cure for patients that cannot be treated with bone marrow transplantation [84,85].

One of the advantages of ex-vivo gene therapy is the ability to ‘sample’ the transduced cells before patient administration. This facilitates efficacy and safety checks before introducing the product to the patient, for example assessing the transduction efficiency and/or the clonality of integration events in the infused product. In the case of ex-vivo gene therapy on cells of the blood, there is also easy access to the transduced cells even after administration via blood sampling. Such approaches are not so straightforward for in-vivo gene therapy where investigators may have to resort to tissue biopsies and disease phenotype assessment to address safety and efficacy, especially in cases where the therapeutic transgene is not secreted into the bloodstream.

For both in-vivo and ex-vivo gene therapy approaches, achieving a high percentage of transduced cells is an important aim. In the ex-vivo setting, transducing as many cells as possible is paramount for the engraftment of transduced CD34^+^ stem cells into the patient’s bone marrow, where a higher proportion of cells expressing the therapeutic gene correlates with higher engraftment success. Aside from the obvious need to achieve a therapeutically relevant dose of expressed protein, transduction efficiency is crucial in-vivo because to induce immune tolerance to a transgene product, a minimal amount of expression is required [86].

Some of the diseases targeted by ex-vivo gene therapy harbor a mutation that causes a proliferative advantage to cells that have been corrected (such as SCID). This will, in time, cause an amplification of cells that are corrected since they will out-proliferate non-corrected cells. Ultimately, as long as this amplification is not unregulated, this advantage is likely to benefit the treated patient. Such an effect is not readily observed in in-vivo lung or liver gene therapy, but examples do exist, and a proliferative advantage can be established artificially. For example, correcting the common PI*Z mutation of AAT deficiency or inhibiting its expression with a microRNA prevents the accumulation of PI*Z polymers in the hepatocytes and give those cells a survival advantage over non-corrected cells that have a tendency to undergo stress-induced apoptosis [87]. A more synthetic approach was taken by Nygaard and colleagues, who developed a vector that, in addition to expressing a therapeutic transgene, also expressed a short hairpin RNA (shRNA) that protects against a toxic drug [88]. Cells that do not have this vector integrated succumb to the drug and allow cells that do harbor the shRNA to outgrow them and at the same time express the therapeutic transgene.

Stem cell gene transfer can be achieved either by ex-vivo or in-vivo gene therapy. This can lead to a life-long cure, where the therapeutic DNA is faithfully retained and expressed in daughter cells, as may be achieved with integrating viral vectors [84]. Whilst in ex-vivo hematopoietic stem cell gene therapy the target cell is evident (e.g. CD34^+^ cells), the same cannot often be said for in-vivo gene therapy for lung or liver. Although there is currently no consensus on the identity of a single ‘lung stem cell’ [26,27], once identified, that stem cell will still require a vector capable of transducing it in an in-vivo setting. The profound differences in developmental and adult lung biology between mice and humans [89] have not made the development of suitable vectors for in-vivo stem cell targeting any easier.

While stem-cell based therapies are inherently attractive, approaches targeting quiescent cells (those that do not readily divide) may also have merit. This is especially true in diseases where the therapeutic gene can give a proliferative advantage, such as SCID, and/or where insertion of an active transcriptional unit (at least a promoter and transgene) can interfere with neighboring genes that perturb the regulation of cell division, leading to either hypo- or hyper-proliferation [81]. It is likely that this insertional mutagenic effect is more profound in cells with high proliferating potential such as stem or progenitor cells. Importantly, approaches to minimize such effects in the ex-vivo field have been widely adopted by those developing vectors for in-vivo gene transfer, including the use of self-inactivating vectors and the increasing move from retroviral to lentiviral vectors.

As the number of gene therapy clinical trials increases there is a consequent need for increased vector manufacturing. Improvement in overall vector production yield and the expansion of manufacturing capacity will be important goals, otherwise these will become bottlenecks to gene therapy progress. This is particularly relevant for in-vivo approaches, where very large quantities of vector may be needed to target whole organs. One problem is that much of the delivered gene therapy agent may be wasted due to inefficient delivery devices, a particular problem for aerosol delivery to the lung and the body’s ability to clear foreign particulate agents [5], an effect that is used to our advantage for liver gene transfer [90]. Crucially, the development of vectors that are more efficient, such that a lower dose is required for efficacy, should also be pursued.

### 3.1. Immune challenges hamper life-long treatment hope

It has been often reported that, in mice, a single dose of a viral vector can last for the lifetime of the animal. Even in larger animal models such as dogs, a single dose can also last for several years without a notable reduction in transgene expression. This finding has not always been reliably translated into the clinic, however, as shown by the pivotal trial by Manno et al [59]. In this trial, it became evident that an unexpectedly strong CD8^+^ cellular immune response to AAV8 capsid proteins remaining in transduced cells was to blame for the short life span of the transduced cells, elevated transaminase levels and ultimately the decline of FIX transgene expression.

Another example, where results from rodent studies do not translate directly to studies with large animal models, is the recent observation that high doses (2e14 GC/kg) of rAAV9 vectors can lead to severe acute toxicity in piglets and non-human primates when delivered intravenously [91,92]. In this case, and different from the trial results reported by Manno et al [59], the toxicity was likely due to an acute, innate immune response (around 5 days after vector administration). The absence of such strong toxicity in the trial reported by Manno et al may be attributed to differences in vector serotype and dose (2e12 GC/kg versus 2e14 GC/kg). However, it is interesting to note that in a recent clinical trial for spinal muscular atrophy similarly large rAAV9 doses (2e14 GC/kg) were administrated intravenously and were well tolerated in a phase 1 [93], now progressing into a phase 3, clinical trial (ClinicalTrials.gov Identifier: NCT03505099). Although caution is warranted and necessitates further investigation, the response needs to be measured in order to put the wellbeing of patients first [94]. If very high vector doses are required for effective therapies there will need to be improved vector potency to lower the dose. Such an approach has been successful for Haemophilia B gene therapy in the use of a high specific-activity transgene and codon-optimized expression cassette leading to lower vector doses with low incidence of anti-capsid immune responses [61].

Even when a deleterious cellular immune response can be avoided, cellular turnover will impact transgene expression duration in liver and lung alike, repeat administration will be required to provide a life-long effect for a disease that, due to its genetic nature, is inherently chronic. Non-clinical and clinical studies in which gene therapy vectors have been repeatedly delivered are not common, repeated systemic and lung administration have so far only been moderately successful for viral vectors. As expected, after systemic injection of a virus the body launches an immune response to the viral proteins capable of neutralizing the virus. Upon a second administration, the immune response will prevent the virus from transducing the cell and often a stronger memory immune response ensues. A similar neutralizing antibody response is typically observed following viral vector delivery to the lung, where rAAV vectors cannot be effectively re-administered [95]. There are several ways proposed to circumvent this problem, including switching of the rAAV serotype and transient immunosuppression [76], although none have so far gained traction in the clinic. Somewhat surprisingly, some configurations of lentiviral vectors can be successfully re-administered to the lung without loss of efficacy, and even show increased expression as a result [96,97]. These vectors hold promise for gene therapy of chronic lung diseases, and understanding the reasons underpinning successful repeat administration could also provide important insight for other vectors.

Although recombinant viruses are the most popular gene delivery vector, with regard to repeat administration the balance is clearly in favor of non-viral gene therapy. Since antibody and cellular immune responses are focused against viral peptides, many non-viral vectors that are based on lipids/polymers and that do not contain peptides, can be safely and effectively repeatedly administered [15].

### 3.2. One-hit-wonder: genome editing

A potential way to achieve a lifelong therapeutic effect for genetic diseases without having to repeat administer is to use genome editing. This process relies on the correction of a mutation directly in the genomic DNA. As genomic DNA is replicated during cell division it will not be diluted out by cell division, as opposed to an episomal non-viral plasmid or rAAV vector. The development of CRISPR/Cas9 has spurred on the genome editing field and a mere 5 years from initial Cas9 studies in cultured cells, the ex-vivo field has commenced clinical trials involving Cas9-mediated genome edited blood cell products in both China and the United States.

However, as discussed in this review, success with ex-vivo approaches does not guarantee success when adopting similar strategies in-vivo. With the first in-vivo gene editing clinical trials also underway (targeting hemophilia B and Mucopolysaccharidosis type I and II), Sangamo Therapeutics is leading the way for in-vivo gene editing. At time of writing, two patients have been injected with their rAAV-based vector and no severe adverse reactions related to the treatment have been reported [98]. The transgene expression approach taken in these studies is unusual, with the integration of the therapeutic transgene targeted to the albumin locus to take advantage of the strong liver albumin promoter activity. In these studies, double strand genomic DNA breakage is achieved via a specific zinc-finger nuclease and homology directed repair is directed by a rAAV encoded DNA donor. Using this approach, high levels of secreted transgene expression are possible even if the percentage of cells that harbor the desired genome modification is low. Time will tell whether this kind of approach will be efficacious enough to achieve therapeutic levels of protein in humans.

A possible roadblock to effective clinical translation of the CRISPR/Cas9 research revolution is the recent finding that a large proportion of humans have pre-existing immunity to two of the widely-used Cas9 orthologues, saCas9 and spCas9 [99]. The antibody reaction found against both orthologues is troubling for the in-vivo use of ribonucleoprotein (RNP) mixtures of recombinant Cas9 protein and synthetic gRNAs without any form of cloaking. However, it is the detection of cytotoxic CD8^+^ T-cells to saCas9 that is potentially more troublesome; these cells are, in principle, capable of eliminating cells that contain saCas9 protein, implying that cells that have been successfully genome edited may be killed off by the patient’s own immune system—effectively decimating any therapeutic benefit. Interestingly, no T-cell reaction was found to spCas9, but more rigorous and sensitive tests are required to confirm this.

## 4. Conclusion

The potential for gene therapy to cure genetic diseases is starting to be realized in both the lung and liver and a comparison of the approaches used in these target organs highlights obstacles and opportunities for both. The clinical trials showing positive efficacy and phenotypic results, combined with the advent of in-vivo gene therapy products (though mainly based on rAAV) being approved by regulatory authorities underscore this [2]. Inhibitory antibody responses to viral vectors remain a challenge, making effective repeat administration to achieve long-term transgene expression elusive. As we describe in this review, there are also pitfalls for in-vivo gene therapy with regard to the ability to target the correct cells, reach the therapeutic threshold of transgene expression, and achieve sufficient duration of expression. To remedy this, it is imperative that the technical development of gene therapy vectors remains a key focus of improvement in the field.

## 5. Expert opinion

In-vivo gene therapy has reached clinical maturity following the approval of gene therapies for use in China, Europe and the USA. Questions remain, however, as to whether the level and duration of transgene expression achieved in animal models will translate to the clinic. On a case by case basis, practitioners will need to carefully consider whether the higher efficiencies of viral vectors outweigh immunological challenges restricting repeated administration. This remains, in our opinion, one of the key factors limiting widespread adoption of in-vivo gene transfer approaches. Several workarounds to mitigate host immunological reactions to repeated administration have been proposed (reviewed [76]), but so far none have gained sufficient traction to warrant the translational step to the clinic. There is one particularly exciting new strategy to allow repeat rAAV dosing, which utilizes rapamycin-loaded nanoparticles delivered simultaneously with the vector, preventing antibody responses to both AAV capsid proteins and the expressed transgene in mice. To-date, this approach has allowed up to three doses of rAAV to be administered without loss of efficacy [100]. We expect that such a combinatorial approach—using small molecules with precise modes of action together with complex biological drugs such as gene transfer vectors—will become more prevalent as the field continues to mature.

An additional hurdle that could be a substantial translational bottleneck, is the extent of good manufacturing process (GMP) grade vector production necessary to support in-vivo gene transfer approaches. A typical clinical development program for in-vivo gene therapy has vector requirements that dwarf those necessary for ex-vivo gene/cell therapy approaches [101]; this is particularly relevant in the lung. This issue is compounded by the observation that many academic and commercial vector manufacturing facilities were designed with scales necessary to support ex-vivo programs in mind [102]. Furthermore, wait times to access such manufacturing facilities appear to be increasing [103]—in part due to spectacular successes achieved with immuno-oncology ex-vivo gene/cell therapy approaches such as chimeric antigen receptor T-cell based therapies [104]. To facilitate the translational success emerging with in-vivo gene transfer, considerable focus must be brought to bear on the cost-of-goods associated with manufacturing vectors. This will require efficient scaling of vector production, for example by developing vector production from stable cell lines [105,106] to reduce the cost and procedural burden of transient transfection that is the backbone of current viral vector approaches [107].

We foresee that development of delivery vectors with improved efficiency and specificity will be combined with improvements in CRISPR/Cas9 technology to make in-vivo genome editing a realistic possibility for specific inherited diseases; and hematopoietic disorders are likely to be the first to benefit. Application to other organs such as the lung and liver will require a deeper understanding of stem cell niche biology to avoid possible transient efficacy resulting from targeting progenitor cell populations rather than true, self-renewing stem cell populations, correction of which might be permanently efficacious. Considering the inherent immunological limitations of viral vectors, non-viral systems have considerable potential to aid translation of genome editing, either through canonical nucleic acids encoding the genome editing machinery, or perhaps more favorably through the use of ribonucleoprotein (RNP) complexes. The inherent ability to repeat administer such non-viral formulations may allow for increased editing efficacy simply by the judicious use of consecutive dosing. Furthermore, the fact that RNPs in particular offer a straightforward approach to transient expression of the genome engineering machinery will, we anticipate, become increasingly more attractive. However, as with the more conventional in-vivo gene therapy, one must remember that delivery to the correct cells is paramount and will ultimately dictate clinical success and market acceptance.

## Article highlights

- In-vivo gene therapies for the lung and liver are reaching clinical maturity, with more treatment modalities reaching the clinic, with gene therapy for hemophilia already approved for use
- Questions about how to maintain and extend transgene expression levels in humans are addressed through the use of novel virus capsids and pseudotypes
- Ex-vivo trailblazing allows important lessons to be learned including exploitation of proliferative advantages
- In-vivo genome editing clinical trials for hemophilia B and MPS I and II are underway, possibly providing the means of life-long effect in a single dose
- Rapamycin loaded nanoparticles may prove the key to repeat administration of AAV vectors

This box summarizes key points contained in the article.
